# Effects of abiotic stressors on lutein production in the green microalga *Dunaliella salina*

**DOI:** 10.1186/1475-2859-13-3

**Published:** 2014-01-08

**Authors:** Weiqi Fu, Giuseppe Paglia, Manuela Magnúsdóttir, Elín A Steinarsdóttir, Steinn Gudmundsson, Bernhard Ø Palsson, Ólafur S Andrésson, Sigurður Brynjólfsson

**Affiliations:** 1 Center for Systems Biology, University of Iceland, Reykjavík 101, Iceland; 2 Department of Bioengineering, University of California, La Jolla, San Diego, CA 92093-0412, U.S.A; 3 Faculty of Industrial Engineering, Mechanical Engineering and Computer Science, University of Iceland, Reykjavík 101, Iceland; 4 Faculty of Life and Environmental Sciences, University of Iceland, Reykjavík 101, Iceland

**Keywords:** *Dunaliella salina*, Adaptive laboratory evolution, Response surface methodology, Lutein production, Osmotic stress, Short-term response

## Abstract

**Background:**

Recent years have witnessed a rising trend in exploring microalgae for valuable carotenoid products as the demand for lutein and many other carotenoids in global markets has increased significantly. In green microalgae lutein is a major carotenoid protecting cellular components from damage incurred by reactive oxygen species under stress conditions. In this study, we investigated the effects of abiotic stressors on lutein accumulation in a strain of the marine microalga *D. salina* which had been selected for growth under stress conditions of combined blue and red lights by adaptive laboratory evolution.

**Results:**

Nitrate concentration, salinity and light quality were selected as three representative influencing factors and their impact on lutein production in batch cultures of *D. salina* was evaluated using response surface analysis. *D. salina* was found to be more tolerant to hyper-osmotic stress than to hypo-osmotic stress which caused serious cell damage and death in a high proportion of cells while hyper-osmotic stress increased the average cell size of *D. salina* only slightly. Two models were developed to explain how lutein productivity depends on the stress factors and for predicting the optimal conditions for lutein productivity. Among the three stress variables for lutein production, stronger interactions were found between nitrate concentration and salinity than between light quality and the other two. The predicted optimal conditions for lutein production were close to the original conditions used for adaptive evolution of *D. salina*. This suggests that the conditions imposed during adaptive evolution may have selected for the growth optima arrived at.

**Conclusions:**

This study shows that systematic evaluation of the relationship between abiotic environmental stresses and lutein biosynthesis can help to decipher the key parameters in obtaining high levels of lutein productivity in *D. salina*. This study may benefit future stress-driven adaptive laboratory evolution experiments and a strategy of applying stress in a step-wise manner can be suggested for a rational design of experiments.

## Background

Photosynthetic microalgae have recently been exploited for the commercial production of foods, feeds and cosmetics, as well as active pharmaceutical ingredients [1-5]. Microalgae have exclusive advantages over higher plants for the sustainable production of both valuable compounds and biomass, since they do not compete with agricultural crops for land. *D. salina* is a model species of green microalgae which has been widely cultivated outdoors for β-carotene production [6]. In a previous study [7] we demonstrated that *D. salina* developed for β-carotene production by adaptive evolution is also a potential producer of lutein under environmental stress conditions in contrast to the original *Dunaliella* strain (UTEX LB #200). Lutein has been widely used as a feed additive and a food coloration agent in industry [8] and it may also protect against age-related macular degeneration in humans [8,9]. Lutein demand in the global market has been increasing rapidly in recent years [8,10]. At present, lutein is mainly produced from the flowers of marigold, but the content is low, 0.3 milligram per gram dry biomass [1]. This has led to considerable interest in other sources of lutein, notably microalgae [8].

Changes in environmental conditions, such as heat shock, nutrient deprivation, osmotic pressure and radiation impose oxidative stress on organisms through the production and accumulation of reactive oxygen intermediates [11]. In adaptation to stress conditions, plants and microalgae show similar patterns of signal transduction, e.g. involving the extracellular signal-regulated kinase (ERK) pathway [12] and generating reactive oxygen species (ROS) as secondary messengers and mediators [13]. Both enzymatic and nonenzymatic antioxidants play important roles in the defense mechanism against oxidative damage, both by scavenging ROS and by inhibiting their generation. Nonenzymatic antioxidants usually refer to ascorbic acid, glutathione, tocopherols, carotenoids and other small molecule antioxidants [11]. Lutein is a major carotenoid in the light harvesting antenna of green algae and higher plants. It plays an important role in harvesting blue light and in transferring energy to the photosystem reaction center, as well as protecting the photosynthetic apparatus against oxidative stress caused by ROS [14]. Lutein is therefore likely to be accumulated in response to different stress conditions involving ROS generation and degradation in cells. However, some stress conditions could exceed the capabilities of *Dunaliella* cells to acclimate, resulting in irreparable damage and cell death instead of adaptation. The original *D. salina* strain (UTEX LB #200) is not suitable for industrial production of lutein since it is sensitive to red light and unable to grow fast at high light intensities, e.g. 170 μE/m^2^/s or higher [7]. We have previously evolved a derivative of *D. salina* UTEX LB #200, named HI 001, which can withstand high light stress and has shown promise as a lutein producer [7]. It is therefore interesting to examine systematically the effects of representative abiotic stressors on the lutein production of *D. salina* HI 001 in batch culture.

**Figure 1:**
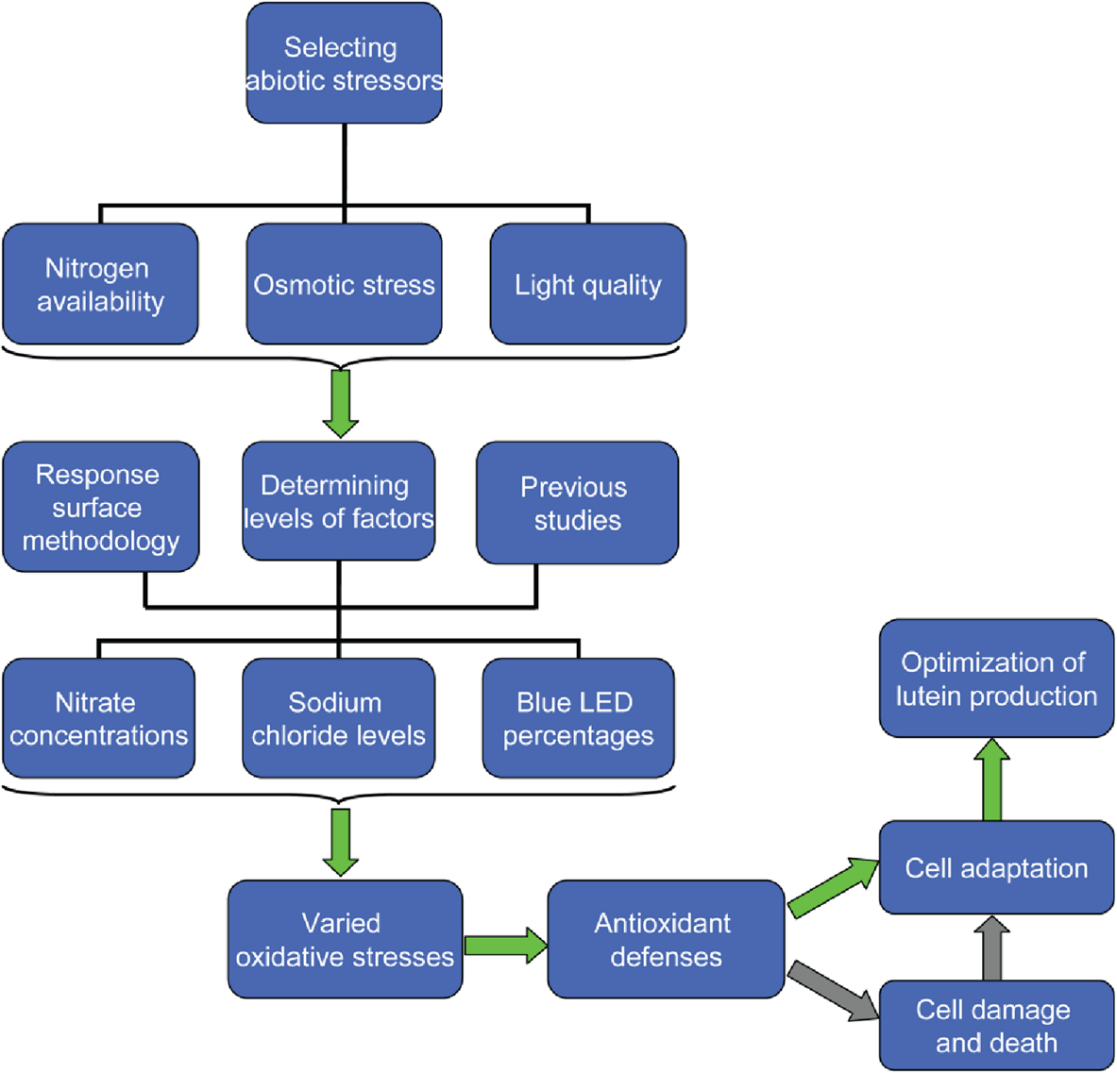
**A schematic design of the study for the optimization of lutein production in *D. salina*.**

Many abiotic stress factors such as irradiance, salinity, and nitrogen deprivation have been widely applied to trigger carotenoid accumulation in *D. salina*[2]. In addition, emerging light-emitting diode (LED) technology makes it possible to study the effects of monochromatic light, e.g. red light, with a narrow spectrum on microalgae [15]. Our previous study suggested that light quality was critical both for *Dunaliella* growth and for carotenoid accumulation [7]. Increasing the photon flux of red LED light alone damaged *Dunaliella* cells (UTEX LB #200) significantly and hindered the accumulation of carotenoids. Combining red LED light with blue LED light allowed growth at a higher total photon flux and the application of adaptive laboratory evolution led to increased accumulation of carotenoids [7]. We have therefore selected light quality, osmotic stress and nitrate concentration as three representative stressors and set out to examine their effects on lutein production in batch cultures of *D. salina* HI 001. Response surface methodology (RSM) is an effective statistical tool used in bioprocess engineering for experimental design, model construction, model validation and process optimization [16-19]. As it is unknown whether the conditions used for adaptive evolution are optimal for lutein production in *D. salina* HI 001. With the aid of RSM, we have set out to study the robustness and flexibility of adaptive evolution for optimizing lutein production in *D. salina* as well as cell adaptability under varied environmental stimuli. The framework of the study design is shown schematically in Figure 1.

## Results

### Response surface experimental design

Nitrogen availability (as indicated by the level of KNO_3_ in the medium), osmotic stress (as indicated by the NaCl level in the medium) and light quality (as indicated by the percentage of the blue LEDs of the total LEDs), were selected as three factors influencing *Dunaliella* growth and associated pigment accumulation. These factors were used as experimental variables in a Box–Behnken type experimental design [20] and the software Design Expert (Stat-Ease Inc., Minneapolis, U.S.A.) was used to analyze the data. A fixed photon flux of 170 μE/m^2^/s was supplied to the PBRs in all the RSM experiments and the center point of the design was chosen as 1.5 M NaCl, 31.2 mM KNO_3_ and 25% blue LEDs. The NaCl concentration was based on previous studies [21,22]. A KNO_3_ concentration of 31.2 mM was previously found to support biomass capacity of 5 gDCW/L [7], and 25% blue LEDs was adopted from our previous study [7]. These growth conditions were previously applied to *D. salina* HI 001 for enhancing growth and carotenoid accumulation through adaptive evolution [7]. Details of the experimental design, including both coded and actual values of the variables are given in Table 1.

**Table 1:** **Coded and actual values of variables in experiments of Box**–**Behnken design**

**Experiment number**	**Coded **^***a ***^**and actual values of variables **^***b***^		
	***X***_***1***_ (%)	***X***_***2***_ (**mM**)	***X***_***3***_ (**M**)
1	0 (-1)	0.2 (-1)	1.5 (0)
2	50 (1)	0.2 (-1)	1.5 (0)
3	0 (-1)	62.2 (1)	1.5 (0)
4	50 (1)	62.2 (1)	1.5 (0)
5	0 (-1)	31.2 (0)	0.5 (-1)
6	50 (1)	31.2 (0)	0.5 (-1)
7	0 (-1)	31.2 (0)	2.5 (1)
8	50 (1)	31.2 (0)	2.5 (1)
9	25 (0)	0.2 (-1)	0.5 (-1)
10	25 (0)	62.2 (1)	0.5 (-1)
11	25 (0)	0.2 (-1)	2.5 (1)
12	25 (0)	62.2 (1)	2.5 (1)
13	25 (0)	31.2 (0)	1.5 (0)
14	25 (0)	31.2 (0)	1.5 (0)
15	25 (0)	31.2 (0)	1.5 (0)

^*a*^ Coded values were in brackets.

^*b*^*X*_*1*_: Blue LED percentage (% of total LEDs); *X*_*2*_: KNO_3_ concentration (mM); *X*_*3*_: NaCl concentration (M).

### Effects of abiotic stressors on growth and lutein production

**Figure 2:**
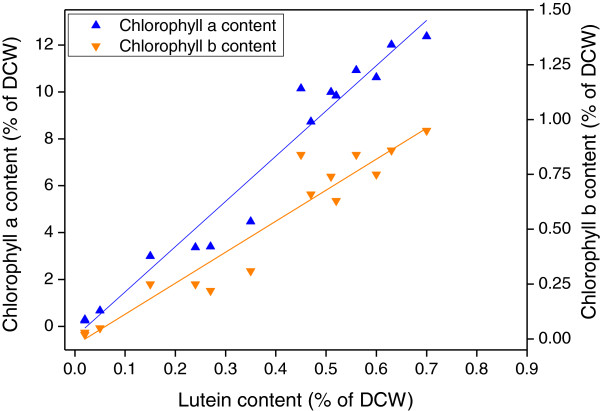
**Correlations between the lutein content and chlorophyll *a *and *b *content in *D. salina *cells (data shown in Table **2**).** Correlation coefficients (Kendall’s tau) were 0.90 and 0.81 for lutein content with chlorophyll *a* content and with chlorophyll *b* content, respectively.

The results of the experiments are shown in Tables 2 and Additional file 1: Table S1. Obvious differences in biomass and lutein productivities as well as chlorophylls and lutein content in cells were observed among the different growth conditions. In addition, lutein accumulation was in good agreement with the chlorophyll *a* and chlorophyll *b* content in *D. salina* (Figure 2). These results suggested that lutein accumulation in *D. salina* was regulated in the same manner as chlorophyll synthesis [7]. The correlation between lutein productivity and biomass productivity further confirmed that lutein was a growth-coupled primary metabolite (Additional file 1: Figure S2).

**Table 2:** **Results **^***a ***^**of design experiments**

**Experiment number**	**Lutein productivity **^***b***^ (**mg**/**L**/**day**)	**Lutein content** (% **of dry biomass** )	**Chlorophyll *a*** (% **of dry biomass**)	**Chlorophyll *b*** (% **of dry biomass**)
1	0.67 ± 0.04	0.27 ± 0.02	3.40 ± 0.26	0.22 ± 0.02
2	0.58 ± 0.01	0.35 ± 0.02	4.47 ± 0.22	0.31 ± 0.01
3	1.35 ± 0.11	0.52 ± 0.02	9.84 ± 0.31	0.63 ± 0.01
4	1.53 ± 0.07	0.60 ± 0.01	10.62 ± 0.21	0.75 ± 0.01
5	0.08 ± 0.02	0.05 ± 0.009	0.67 ± 0.08	0.05 ± 0.004
6	0.18 ± 0.02	0.15 ± 0.03	2.99 ± 0.54	0.25 ± 0.04
7	1.54 ± 0.01	0.63 ± 0.03	12.01 ± 0.49	0.86 ± 0.04
8	1.16 ± 0.01	0.47 ± 0.04	8.73 ± 0.22	0.66 ± 0.04
9	0.02 ± 0.01	0.02 ± 0.003	0.25 ± 0.04	0.02 ± 0.004
10	0	0.02 ± 0.003	0.29 ± 0.02	0.03 ± 0.005
11	0.44 ± 0.004	0.24 ± 0.02	3.37 ± 0.38	0.25 ± 0.03
12	1.22 ± 0.15	0.45 ± 0.04	10.14 ± 0.21	0.84 ± 0.01
13	2.71 ± 0.18	0.56 ± 0.03	10.92 ± 0.26	0.84 ± 0.05
14	3.45 ± 0.37	0.70 ± 0.07	12.36 ± 0.44	0.95 ± 0.02
15	2.43 ± 0.15	0.51 ± 0.04	9.99 ± 0.13	0.74 ± 0.03

^*a*^ Values were averaged from three independent experiments (mean ± SD).

^*b*^ Lutein productivity was calculated by multiplying lutein content by biomass productivity (see Additional file 1: Table S1).

The following quadratic model was obtained after averaging the triplicate measurements (resulting in 15 data points available for model estimation).

(1) $$\begin{array}{ll} Y= & –2.9112+0.0639X_{1}+0.0676X_{2}+4.5330X_{3}+0.000087X_{1}X_{2}\\ & –0.0048X_{1}X_{3}–0.0065X_{2}X_{3}–0.0012{X_{1}}^{2}–0.0011{X_{2}}^{2}–1.3682{X_{3}}^{2} \end{array}$$

where *Y* is the daily lutein productivity (mg/L/day), *X*_*1*_ is the percentage of blue LED (% of total), *X*_*2*_ is the KNO_3_ concentration (mM) and *X*_*3*_ is the NaCl concentration (M) in the medium. The model in coded values is given by Additional file 1: Equation S1.

**Figure 3:**
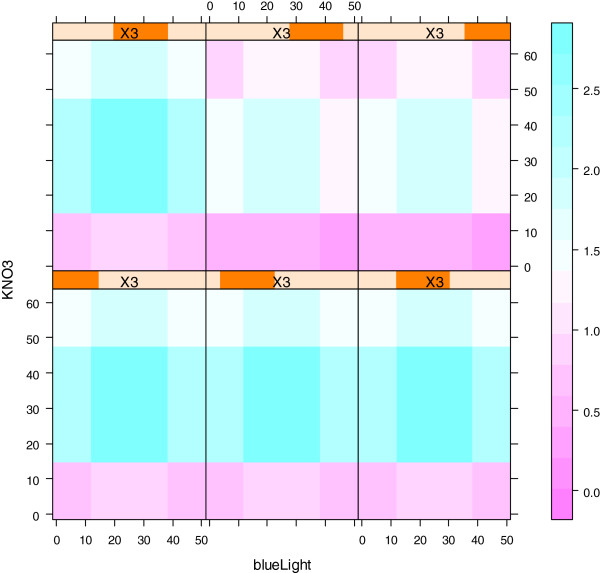
**Evaluation of abiotic stressors on lutein production using a boosted trees model.** Each of the contour plots shows lutein productivity as a function of KNO_3_ (mM) levels and blue LED percentage for fixed levels of NaCl. Purple represents low productivity and cyan represents high productivity. The NaCl levels are indicated by X3 (from low to high). The predictive model is piecewise linear which results in a rectangular partition of the variable space.

The quadratic model was used to predict optimal conditions for lutein production. For the tree-based model, all 3»15 = 45 data points were used. This model was then used to study the effects of each of the three variables on lutein production (Figure 3). The model predicts that the highest levels of lutein are achieved close to the center point of the experiment (Figure 3, bottom-right most plot). Comparison of the three variables in terms of their relative influence on lutein production levels showed that NaCl has the greatest influence, followed by KNO_3_ and the percentage of blue LED has the least influence (data not shown). The strongest variable interactions were between KNO_3_ and NaCl levels while the interaction strength between the percentage of blue LED light with the two other variables was considerably lower (Additional file 1: Table S2).

### Adaptation of *D. salina* to osmotic stress

It is important to test the capability of *D. salina* to regain optimal growth in face of changing environmental conditions since lutein production was found to be growth-coupled (Additional file 1: Figure S2). It was found that osmotic stress, especially hypo-osmotic stress, led to extremely low lutein productivity as well as low chlorophyll *a* content in *D. salina* (Tables 2 and Additional file 1: Table S1, and Figure 2). Comparisons between the values predicted by the quadratic model (Equation 1) and the experimental data (Additional file 1: Table S3) revealed that the model has relatively low prediction accuracy for the hypo-osmotic stress conditions. This was also the case for the tree-based model (data not shown). We conjecture that *D. salina* is sensitive to hypo-osmotic stress and that it might fail to adapt to such osmotic changes. Previous studies have found that *D. salina* is capable of thriving in NaCl solutions between 0.05 M to 5.5 M [23]. However, the sensitivity or tolerance of *D. salina* to hyper-osmotic and hypo-osmotic changes has not been examined, to the best of our knowledge.

**Figure 4:**
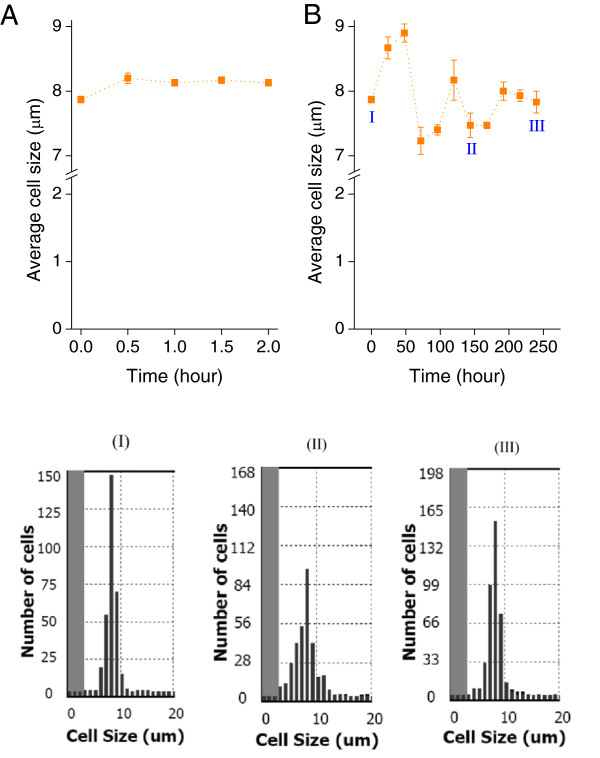
**Average cell sizes and their schematic distributions during *D. salina *response after hypo**-**osmotic shock.***D. salina*: immediate response over the first two hours **(A)** and pre-adaptation over ten days **(B)**; cell size distribution at 0 h **(I)**, 144 h **(II)**, and 240 h **(III)**. *D. salina* cells were cultivated in Gg-8 medium containing 1.5 M NaCl for five days and then the concentrated cells were transferred to Gg-8 medium containing 0.5 M NaCl. The cell size values are averaged from three independent experiments. The error bars indicate the standard deviation.

**Figure 5:**
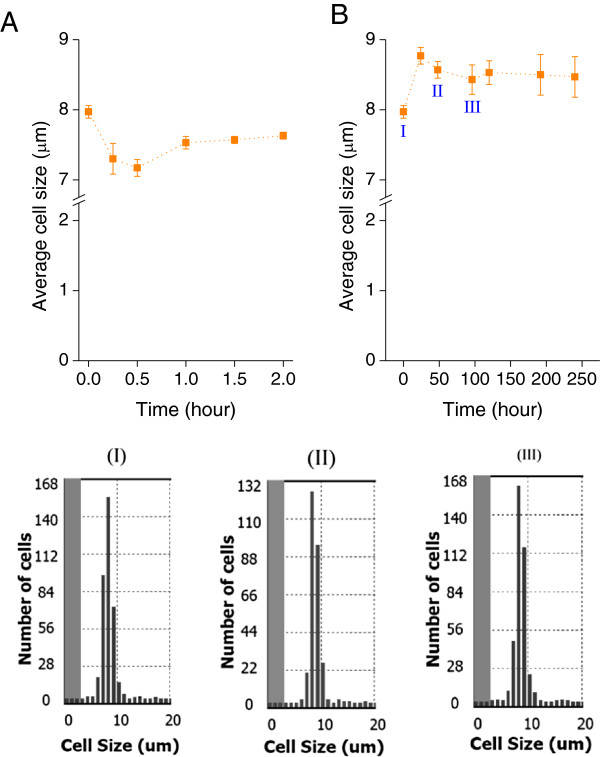
**Average cell sizes and their schematic distributions during *D. salina *response after hyper**-**osmotic shock.***D. salina*: immediate response over the first two hours **(A)** and pre-adaptation over ten days **(B)**; cell size distributions at 0 h **(I)**, at 48 h **(II)**, and at 96 h **(III)**. *D. salina* cells were cultivated in Gg-8 medium containing 1.5 M NaCl for five days and then concentrated cells were transferred to Gg-8 medium containing 2.5 M NaCl. The average cell size values are averaged from three independent experiments. The error bars indicate the standard deviation.

To determine morphological responses of *D. salina* responds to osmotic changes, we measured the cell size for ten days under both hypo-osmotic and hyper-osmotic conditions (Figures 4 and 5). The cell size was distributed mainly between 7.0 μm and 11.0 μm initially (at 0 h). The cells were usually oval in shape rather than spherical and the average cell size was 8.0 μm (Figures 4-I and 5-I). After a hypo-osmotic shift, the *D. salina* cells changed their volume rapidly and the average cell size increased to 9.0 μm at 48 h. Two similar cycles of increase and decrease in average cell size were observed from 24 h to 192 h (Figure 4B) and revealed that the cells were experiencing serious swelling (increasing cell size), cell burst and death (decreasing cell size). The cell size then stabilized after 192 h (Figure 4B).

In contrast, after a hyper-osmotic shift, average cell size decreased immediately to 7.2 μm at 0.5 h and increased to 8.8 μm at 24 h. Average cell size then decreased gradually to 8.4 μm and stabilized in ten days (Figure 5B). The cell size distributions over the time course were unchanged, indicating that there was no significant cell damage. It appears that *D. salina* cells are more tolerant to hyper-osmotic stress than to hypo-osmotic stress.

To summarize, hypo-osmotic stress caused significant changes of cell size distributions and average cell size for 192 h (Figure 4) upon osmotic shift while hyper-osmotic stress just slightly increased the average cell size of *D. salina* (Figure 5). These results indicated that *D. salina* had difficulties adapting to the hypo-osmotic shift with substantial die-off due to irreparable damages when the imposed stress exceeded the capabilities of *Dunaliella* cells to acclimate.

### Prediction of optimal conditions for lutein production

The quadratic model was used to predict the optimal conditions for lutein production (Additional file 1: Table S4). We then conducted three independent experiments in the same PBR system by setting the levels of the variables to the optimal values predicted by the model: blue LED 24.4% of total; nitrogen concentration 36.0 mM; NaCl concentration 1.7 M. This set of values also corresponds to the region of maximum lutein production for the tree-based model (bottom-right most plot in Figure 3). The resulting lutein productivity was 3.68 ± 0.44 mg/L/day and the lutein content was 8.87 ± 1.31 mg/gDCW. This shows that the models are useful for predicting the optimal conditions for lutein production. The conditions predicted by the model are similar to the conditions obtained by adaptive laboratory evolution (Additional file 1: Table S5) as indicated by the small differences in variable values as well as in lutein productivity. Combined with the observation that lutein productivity is positively correlated with biomass productivity, these results indicate that the previous conditions used for adaptive evolution restrict the space of optimal conditions for growth-coupled metabolite production in *D. salina*.

## Discussion

Microalgae have attracted considerable attention recently as they have potential as platform sources in the bio-based industry. This study has provided new data on the production of lutein using photosynthetic microalgae. As the original *D. salina* (UTEX LB #200) is unable to grow fast under red light at high intensities, e.g. 170 μE/m^2^/s, and is therefore not suitable for industrial applications, the *Dunaliella* strain HI 001 previously derived by ALE treatment was studied further in connection to lutein production. We modeled the dependence of lutein productivity on the percentage of blue LEDs of total LED illumination, as well as KNO_3_ and NaCl levels in the medium. A study of the response of *D. salina* to osmotic stress revealed that excessive stress induced by hypo-osmotic changes led to serious cell damage and death rather than adaptation. By utilizing the optimal conditions predicted by quadratic modeling, the productivity achieved was 3.68 ± 0.44 mg/L/day with a lutein content of 8.87 ± 1.31 mg/gDCW. The high similarity between the model optimum for lutein production and the conditions in which *Dunaliella* strain HI 001 had undergone ALE treatment, suggests that the conditions used for adaptive evolution had influenced the optimum arrived at in growth-coupled lutein production by batch cultures of the HI 001 strain.

Several abiotic stress factors are known to inhibit growth in higher plants as well as in microalgae [12]. In response to unfavorable conditions, higher plants and microalgae generate reactive oxygen species (ROS) leading to adaptation by initiation of a phosphorylation cascade and activation of major stress-response genes [24]. Under hyper-osmotic conditions, *Dunaliella* most probably responds by adjusting the concentration of intracellular compatible solutes, primarily glycerol, decreasing the trans-membrane osmotic gradient caused by the high extracellular NaCl concentration [23,25,26]. In this study, salinity-induced osmotic stress played an important physiological role in the *Dunaliella* cells. Hyper-osmotic stress (extracellular NaCl increasing from 1.5 M to 2.5 M) led to salt tolerance of *Dunaliella*, most likely by up-regulating the glycerol metabolism (Figures 5 and Additional file 1: Table S5) while hypo-osmotic stress (extracellular NaCl decreasing from 1.5 M to 0.5 M) damaged cells and led to significant cell death (Figures 4 and Additional file 1: Table S4). It has been reported that hypo-osmotic stress inhibits enzyme activities and expression levels of carbonic anhydrase accompanied by significant induction of ROS production in *D. salina* and consequently algal photosynthesis and growth are suppressed [27]. Lesser [11] also suggested that hypo-osmotic stress led to ROS-induced programmed cell death.

Adaptive laboratory evolution [28,29] has proven successful in developing microbes with improved fitness to specific conditions and increased tolerance to environmental stresses. Since the antioxidant lutein is functional in the detoxification of the ROS produced [14] and its production is also growth-coupled, stress-driven adaptation is highly important for lutein production in microalgae. However, extreme stress can lead to adverse consequences as shown in our previous study [7]. When excess stress was imposed by red light at high intensity, cells failed to acclimate, and an alternative strategies, i.e. partly replacing the red light with blue light, was adopted and found to be beneficial to cell adaptation at the same light intensity [7]. Interestingly, after experiencing ALE under combined blue and red light conditions *D. salina* gained enhanced light tolerance under red light only conditions at the same total photon flux of 170 μE/m^2^/s [7]. It has also been found that blue light is necessary in diatoms for photoacclimation to high light intensities [30]. These phenomena confirm the importance of studying the effects of varying environmental stimuli systematically, since microalgae have developed varying capabilities in acclimating to different stress factors during natural evolution. Furthermore, the percentage of blue LED has limited influence on lutein productivity. As the *D. salina* HI 001 strain had already gained enhanced tolerance to red LED illumination (Additional file 1: Figure S1), it is expected that providing nonlethal stress with either red LED or combined blue and red LED illumination would result in increased lutein accumulation in cells. It should also be noted that the original *D. salina* strain UTEX LB #200 was recognized and suggested as *D. viridis* based on its morphological and biochemical characters [31] while it was grouped with *D. pseudosalina* CONC 010 on the basis of molecular data [32]. As lutein is the main carotenoid produced by *D. viridis*[31], the strain *D. salina* HI 001 used in this study, a derivative of strain UTEX LB #200 is very likely also a good lutein producer.

## Conclusions

Systematic evaluation of the relationship between abiotic environmental stresses and lutein biosynthesis helped to determine the key impact factors and yield high levels of lutein productivity in *D. salina*. Assessment of stress conditions revealed that *Dunaliella* cells displayed varying adaptations to different environmental changes. This study suggests a new guideline for future stress-driven adaptive evolution experiments and a strategy of applying stress in a step-wise manner can be proposed for rational design of experiments.

## Materials and methods

### Microalga and growth conditions

*D. salina* strain was originally obtained from the University of Texas at Austin (UTEX LB #200) and developed by adaptive laboratory evolution (ALE) by means of a semi-continuous culture system with repeated five day cycles [7]. Specifically, *D. salina* after ALE treatment, referred to as HI 001, was successfully cultivated under 170 μE/m^2^/s of red LED light (Additional file 1: Figure S1) [7]. Culture pH of all experiments was maintained between 6.5 and 7.5 by the buffer systems in the medium. For the RSM experiments, seed cultures of *D. salina* cells (HI 001) were grown in Gg-8 medium under the same conditions as the previous ALE treatment, i.e. a total photon flux of 170 μE/m^2^/s consisting of blue LED (42 μE/m^2^/s) and red LED (128 μE/m^2^/s) lights until late exponential phase and then used for subsequent experiments. For all the RSM experiments, *D. salina* was cultivated in batch culture for 5 days under different light conditions with a fixed total photon flux of 170 μE/m^2^/s and a Gg-8 medium which was modified in order to obtain different levels of NaCl and KNO_3_. Detailed growth conditions for all the RSM experiments are shown in Table 1. The biomass concentration during batch culture for all the experiments was relatively high (*A*_660nm_ ≥ 1.0) and the supplied light, as measured on the inner surface of the PBR, was assumed to be all absorbed by the *D. salina* cells [7]. All the experiments were performed in triplicates.

### Parameters for the photobioreactors

Cylindrical bubble column photobioreactors with H = 30 cm, D = 4.0 cm, and a working volume of 300 ± 5 ml [15] were used. The input gas level was 90 ml/min of 2.5% CO_2_ in air.

### Artificial light supply and setup

Blue (Part number: VAOL-5LSBY2) and red (Part number: SSL-LX5093SRC) LED arrays with narrow output spectra (20 nm bandwidth at half peak height) of 470 ± 20 nm and 660 ± 20 nm, respectively, were purchased from LUMEX Inc. (Taiwan, China). The photon flux of the light supplied to the PBRs was measured on the inner surface of each PBR by using a quantum sensor (SR. NO. Q40526 of QUANTUM, Model LI-1400, LI-COR biosciences, Lincoln, Nebraska, U.S.A.). For this study, average photon flux was fixed at 170 μE/m^2^/s by using the duty cycles at a frequency of 10 kHz of flashing light [15,33].

### Adaptation of *D. salina* to osmotics stress

For the adaptation study, *D. salina* cells were first adapted to Gg-8 medium under a total photon flux of 170 μE/m^2^/s red light for five days and used as seed culture. Cell pellets of seed culture were then harvested by centrifugation (1000 × *g* for 10 min) and cultivated in two modified Gg-8 media, i.e. Gg-8 medium containing 2.5 M NaCl for the hyper-osmotic stress study and Gg-8 medium containing 0.5 M NaCl for the hypo-osmotic stress study, respectively.

### Biomass determination

Alga samples of culture suspension were filtered and collected on a mixed cellulose membrane (pore size: 0.45 μm), washed with de-ionized water twice and dried overnight at 60°C before weighing [7].

### Determination of cell size

The cell size was measured by a Countess automated cell counter (Life Technologies Corporation, Carlsbad, California, U.S.A.). *D. salina* cell size was detected in bead mode without using trypan blue dye staining.

### Chlorophyll and carotenoid analysis

The cell pellets were collected by centrifugation (1000 × *g* for 10 min) at 4°C and then extracted with 3ml of ethanol: hexane 2:1 (*v*/*v*) containing 0.1% (*w*/*v*) butylated hydroxytoluene till colorless [34]. To the mixed solution, 2 ml de-ionized water and 4 ml hexane were added and the mixture was vigorously shaken and centrifuged again at 1000 × *g* for 5 min [7]. An aliquot of 4 ml of the upper hexane layer was evaporated under nitrogen at 25 ± 2°C, reconstituted in a mixture of methyl tertiary butyl ether : acetonitrile (50:50) and analyzed by ultra- performance liquid chromatography, UV and mass spectrometry detection (UPLC-UV-MS) according to the procedures described previously [35].

### Modeling approaches for simulations and predictions

Two types of models were created for studying the effects of light quality, nitrogen availability and osmotic stress on lutein productivity (the response variable). The predictor variables were the percentage of blue light, the amount of KNO_3_ and the amount of NaCl. The first model was a traditional quadratic model where the model parameters were obtained with least squares regression. The second model was a nonparametric model, meaning that no assumptions are made about the data generating mechanism. The model was based on gradient boosted regression trees which have received considerable attention in recent years for their superior predictive performance and their usefulness in data exploration [36]. The boosted tree model was obtained with the GBM package for R [37]. The GBM parameters were set as follows: Squared error loss was used, the number of trees was 700 (determined by minimizing the out-of-bag error), shrinkage was set to 0.005, the subsampling fraction to 0.5 and three-way interactions were used.

## Abbreviations

ALE: Adaptive laboratory evolution; ERK: Extracellular signal-regulated kinase; LED: Light-emitting diode; PBRs: Photobioreactors; ROS: Reactive oxygen species; RSM: Response surface methodology; UPLC-UV-MS: Ultra-performance liquid chromatography, UV and mass spectrometry.

## Competing interests

The authors declare they have no competing interests.

## Authors’ contributions

WF designed the study, carried out most of the experiments and wrote the manuscript. GP and MM assisted in analysis of algal samples with LC/MS. EAS participated in some growth experiments. SG participated in modelling work. SG, OSA and SB contributed to editing and revising the manuscript. BØP and SB conceived of the study and helped to finalize the manuscript. All authors read and approved the manuscript.

## Supplementary Material

Additional file 1: Figure S1 Linear growth of adapted *D. salina* (HI 001) under a total light intensity of 170 μE/m^2^/s red LED light. **Figure S2** Correlation between lutein productivity and biomass productivity of *D. salina* cells (data shown in Table 2 and Table S1). **Table S1** Biomass productivity of *D. salina* in RSM experiments ^*a*^, **Table S2** Strength of variable interactions for the boosted tree model (higher values indicate more strength), **Table S3** Comparisons between values predicted by the quadratic model and the experimental data, **Table S4** Prediction of maximum lutein productivity by the quadratic model, **Table S5** Comparison of optimal conditions predicted for lutein production by RSM and conditions developed for carotenoids production by previous ALE. Quadratic model in coded values (**Equation S1**). Click here for file

## References

[B1] Cordero BF Obraztsova I Couso I Leon R Vargas MA Rodriguez H Enhancement of lutein production in *Chlorella sorokiniana* (chlorophyta) by improvement of culture conditions and random mutagenesis Mar Drugs 2011 9 1607 1624 10.3390/md9091607 22131961 PMC3225938

[B2] Lamers PP Janssen M De Vos RCH Bino RJ Wijffels RH Exploring and exploiting carotenoid accumulation in *Dunaliella salina* for cell-factory applications Trends Biotechnol 2008 26 631 638 10.1016/j.tibtech.2008.07.002 18752860

[B3] Pulz O Gross W Valuable products from biotechnology of microalgae Appl Microbiol Biotechnol 2004 65 635 648 10.1007/s00253-004-1647-x 15300417

[B4] Suh IS Joo HN Lee CG A novel double-layered photobioreactor for simultaneous Haematococcus pluvialis cell growth and astaxanthin accumulation J Biotechnol 2006 125 540 546 10.1016/j.jbiotec.2006.03.027 16647774

[B5] Vilchez C Forjan E Cuaresma M Bedmar F Garbayo I Vega JM Marine carotenoids: biological functions and commercial applications Mar Drugs 2011 9 319 333 10.3390/md9030319 21556162 PMC3083653

[B6] Del Campo JA Garcia-Gonzalez M Guerrero MG Outdoor cultivation of microalgae for carotenoid production: Current state and perspectives Appl Microbiol Biotechnol 2007 74 1163 1174 10.1007/s00253-007-0844-9 17277962

[B7] Fu W Gudmundsson O Paglia G Herjolfsson G Andrésson OS Palsson BØ Brynjolfsson S Enhancement of carotenoid biosynthesis in the green microalga *Dunaliella salina* with light-emitting diodes and adaptive laboratory evolution Appl Microbiol Biotechnol 2013 97 2395 2403 10.1007/s00253-012-4502-5 23095941 PMC3586100

[B8] Fernandez-Sevilla JM Acien Fernandez FG Molina Grima E Biotechnological production of lutein and its applications Appl Microbiol Biotechnol 2010 86 27 40 10.1007/s00253-009-2420-y 20091305

[B9] Carpentier S Knaus M Suh M Associations between lutein, zeaxanthin, and age-related macular degeneration: an overview Crit Rev Food Sci Nutr 2009 49 313 326 10.1080/10408390802066979 19234943

[B10] Blanco AM Moreno J Del Campo JA Rivas J Guerrero MG Outdoor cultivation of lutein-rich cells of *Muriellopsis sp*. in open ponds Appl Microbiol Biotechnol 2007 73 1259 1266 10.1007/s00253-006-0598-9 17033775

[B11] Lesser MP Oxidative stress in marine environments: Biochemistry and physiological ecology Annu Rev Physiol 2006 68 253 78 10.1146/annurev.physiol.68.040104.110001 16460273

[B12] Jimenez C Cossio BR Rivard CJ Berl T Capasso JM Cell division in the unicellular microalga *Dunaliella viridis* depends on phosphorylation of extracellular signal-regulated kinases (ERKs) J Exp Bot 2007 58 1001 1011 10.1093/jxb/erl260 17220513

[B13] Apel K Hirt H Reactive oxygen species: metabolism, oxidative stress, and signal transduction Annu Rev Plant Biol 2004 55 373 399 10.1146/annurev.arplant.55.031903.141701 15377225

[B14] Jahns P Holzwarth AR The role of the xanthophyll cycle and of lutein in photoprotection of photosystem II Biochim Biophys Acta 1817 2012 182 193 10.1016/j.bbabio.2011.04.012 21565154

[B15] Fu W Gudmundsson O Feist AM Herjolfsson G Brynjolfsson S Palsson BØ Maximizing biomass productivity and cell density of *Chlorella vulgaris* by using light-emitting diode-based photobioreactor J Biotechnol 2012 161 242 249 10.1016/j.jbiotec.2012.07.004 22796827

[B16] Ghosalkar A Sahai V Srivastava A Optimization of chemically defined medium for recombinant Pichia pastoris for biomass production Bioresour Technol 2008 99 7906 7910 10.1016/j.biortech.2008.01.059 18325760

[B17] Holmes WJ Darby RAJ Wilks MDB Smith R Bill RM Developing a scalable model of recombinant protein yield from *Pichia pastoris*: the influence of culture conditions, biomass and induction regime Microb Cell Fact 2009 8 35 10.1186/1475-2859-8-35 19570229 PMC2717918

[B18] Jafari R Sundström BE Holm P Optimization of production of the anti-keratin 8 single-chain Fv TS1-218 in *Pichia pastoris* using design of experiments Microb Cell Fact 2011 10 34 10.1186/1475-2859-10-34 21575236 PMC3119184

[B19] Zhi W Song J Ouyang F Bi J Application of response surface methodology to the modeling of α-amylase purification by aqueous two-phase systems J Biotechnol 2005 118 157 165 10.1016/j.jbiotec.2005.03.017 15955584

[B20] Box G Behnken D Some new three level designs for the study of quantitative variables Technometrics 1960 2 455 475 10.1080/00401706.1960.10489912

[B21] Shaish A Ben-Amotz A Avron M Biosynthesis of β-carotene in *Dunaliella* Methods Enzymol 1992 213 439 444

[B22] Jahnke LS Massive carotenoid accumulation in *Dunaliella bardawil* induced by ultraviolet-A radiation J Photochem Photobiol B: Biol 1999 48 68 74 10.1016/S1011-1344(99)00012-3

[B23] Chen H Jiang JG Osmotic responses of *Dunaliella* to the changes of salinity J Cell Physiol 2009 219 251 258 10.1002/jcp.21715 19202552

[B24] Zuppini A Gerotto C Baldan B Programmed cell death and adaptation: Two different types of abiotic stress response in a unicellular chlorophyte Plant Cell Physiol 2010 51 884 895 10.1093/pcp/pcq069 20457671

[B25] Ben-Amotz A Adaptation of the unicellular alga *Dunaliella parva* to a saline environment J Phycol 1975 11 50 54

[B26] Ben-Amotz A Avron M The role of glycerol in osmotic regulation of the halophilic alga *Dunaliella parva* Plant Physiol 1973 51 875 878 10.1104/pp.51.5.875 16658431 PMC366367

[B27] Liu W Ming Y Li P Huang Z Inhibitory effects of hypo-osmotic stress on extracellular carbonic anhydrase and photosynthetic efficiency of green alga Dunaliella salina possibly through reactive oxygen species formation Plant Physiol Biochem 2012 54 43 48 22377429 10.1016/j.plaphy.2012.01.018

[B28] Palsson BØ Adaptive laboratory evolution Microbe 2011 6 69 74

[B29] Conrad TM Lewis NE Palsson BØ Microbial laboratory evolution in the era of genome-scale science Mol Syst Biol 2011 7 509 21734648 10.1038/msb.2011.42 PMC3159978

[B30] Schellenberger Costa B Jungandreas A Jakob T Weisheit W Mittag M Wilhelm C Blue light is essential for high light acclimation and photoprotection in the diatom *Phaeodactylum tricornutum* J Exp Bot 2013 64 483 493 10.1093/jxb/ers340 23183259 PMC3542041

[B31] Borowitzka MJ Siva CJ The taxonomy of the genus *Dunaliella* (Chlorophyta, Dunaliellales) with emphasis on the marine and halophilic species J Appl Phycol 2007 19 567 590 10.1007/s10811-007-9171-x

[B32] González MA Coleman AW Gómez PI Montoya R Phylogenetic relashionship among strains of *Dunaliella* (Chlorophyceae) based on nuclear ITS rDNA sequences J Phycol 2001 37 604 611 10.1046/j.1529-8817.2001.037004604.x

[B33] Park KH Lee CG Effectiveness of flashing light for increasing photosynthetic efficiency of microalgal cultures over a critical cell density Biotechnol Bioprocess Eng 2001 6 189 193 10.1007/BF02932549

[B34] Garcia-Gonzalez M Moreno J Manzano JC Florencio FJ Guerrero MG Production of *Dunaliella salina* biomass rich in 9-*cis*-β-carotene and lutein in a closed tubular photobioreactor J Biotechnol 2005 115 81 90 10.1016/j.jbiotec.2004.07.010 15607227

[B35] Fu W Magnusdottir M Brynjolfson S Palsson BØ Paglia G UPLC-UV-MS^E^ method for quantification and identification of major carotenoid and chlorophyll species in algae Anal Bioanal Chem 2012 404 3145 3154 10.1007/s00216-012-6434-4 23052878

[B36] Hastie T Tibshirani R Friedman J The elements of statistical learning: data mining, inference, and prediction 2009 Heidelberg: Springer

[B37] Ridgeway Ggbm: Generalized boosted regression models. R package version 2.1 2006 http://cran.r-project.org/web/packages/gbm

