# Supplementary material for: Effects of abiotic stressors on lutein production in the green microalga Dunaliella salina
Source: Microb Cell Fact. 2014 Jan 8;13:3. doi: 10.1186/1475-2859-13-3 (PMC3893366; doi:10.1186/1475-2859-13-3)
Supplement: Additional file 1: Figure S1 — Linear growth of adapted D. salina (HI 001) under a total light intensity of 170 μE/m2/s red LED light. Figure S2 Correlation between lutein productivity and biomass productivity of D. salina cells (data shown in Table 2 and Table S1). Table S1 Biomass productivity of D. salina in RSM experiments a , Table S2 Strength of variable interactions for the boosted tree model (higher values indicate more strength), Table S3 Comparisons between values predicted by the quadratic model and the experimental data, Table S4 Prediction of maximum lutein productivity by the quadratic model, Table S5 Comparison of optimal conditions predicted for lutein production by RSM and conditions developed for carotenoids production by previous ALE. Quadratic model in coded values (Equation S1). [file 1475-2859-13-3-S1.doc]

**This supplementary document includes two figures, five tables and one equation.**

**Fig. S1** Linear growth of adapted *D. salina* (HI 001) under a total light intensity of 170 µE/m^2^/s red LED light. M2-1, M2-2 and M2-3 represent three independent experiments.

**Fig. S2** Correlation between lutein productivity and biomass productivity of *D. salina* cells (data shown in Table 2 and Table S1). Correlation coefficient (Kendall’s tau) is 0.76.

**Table S1** Biomass productivity of *D. salina* in RSM experiments *^a^*

| Experiment number | Biomass productivity (gDCW/L/day) |
| --- | --- |
| 1 | 0.25±0.01 |
| 2 | 0.17±0.01 |
| 3 | 0.26±0.01 |
| 4 | 0.25±0.01 |
| 5 | 0.17±0.01 |
| 6 | 0.12±0.01 |
| 7 | 0.24±0.01 |
| 8 | 0.25±0.002 |
| 9 | 0.13±0.02 |
| 10 | -0.01±0.004 |
| 11 | 0.18±0.01 |
| 12 | 0.27±0.01 |
| 13 | 0.48±0.02 |
| 14 | 0.50±0.01 |
| 15 | 0.48±0.02 |

*^a^* Values were averaged from three independent experiments (mean±SD).

**Table S2 Strength of variable interactions for the boosted tree model (higher values indicate more strength)**

|  | KNO_3_ | NaCl |
| --- | --- | --- |
| Blue LED | 0.10 | 0.11 |
| KNO_3_ |  | 0.29 |

**Table** **S3** Comparisons between values predicted by the quadratic model and the experimental data

| Experiment number | *X*_1_ (%) | *X*_2_ (mM) | *X*_3_ (M) | *Y* (predicted) | *Y* (measured) | Value difference |
| --- | --- | --- | --- | --- | --- | --- |
| 1 | 0 | 0.2 | 1.5 | 0.83 | 0.67 | 0.16 |
| 2 | 50 | 0.2 | 1.5 | 0.66 | 0.58 | 0.08 |
| 3 | 0 | 62.2 | 1.5 | 1.37 | 1.35 | 0.02 |
| 4 | 50 | 62.2 | 1.5 | 1.47 | 1.53 | 0.06 |
| 5 | 0 | 31.2 | **0.5** | 0.15 | 0.08 | 0.07 |
| 6 | 50 | 31.2 | **0.5** | 0.36 | 0.18 | 0.18 |
| 7 | 0 | 31.2 | 2.5 | 1.42 | 1.54 | 0.12 |
| 8 | 50 | 31.2 | 2.5 | 1.15 | 1.16 | 0.01 |
| 9 | 25 | 0.2 | **0.5** | -0.18 | 0.02 | 0.20 |
| 10 | 25 | 62.2 | **0.5** | 0.09 | -0.002 | 0.09 |
| 11 | 25 | 0.2 | 2.5 | 0.43 | 0.44 | 0.01 |
| 12 | 25 | 62.2 | 2.5 | 1.51 | 1.22 | 0.29 |
| 13 | 25 | 31.2 | 1.5 | 2.89 | 2.71 | 0.18 |
| 14 | 25 | 31.2 | 1.5 | 2.89 | 3.45 | 0.56 |
| 15 | 25 | 31.2 | 1.5 | 2.89 | 2.43 | 0.46 |

**Table S4** Prediction of maximum lutein productivity by the quadratic model

|  | Goal and constraints | Lower limit | Upper limit | Solution number 1 |
| --- | --- | --- | --- | --- |
| *X_1_*: Blue LED percentage (% of total LEDs) | is in range | 0 | 50 | 24.4 |
| *X_2_*: Nitrogen concentration (mM) | is in range | 0.2 | 62.2 | 36.0 |
| *X_3_*: NaCl concentration (M) | is in range | 0.5 | 2.5 | 1.7 |
| Lutein productivity (mg/L/day) | maximize | -0.002 | 3.45 | 2.97 |

**Table S5** Comparison of optimal conditions predicted for lutein production by RSM and conditions developed for carotenoids production by previous ALE

|  | Levels | |
| --- | --- | --- |
| Variables | RSM | ALE |
| *X_1_*: Blue LED percentage (% of total LEDs) | 24.4 | 25.0 |
| *X_2_*: Nitrogen concentration (mM) | 36.0 | 31.2 |
| *X_3_*: NaCl concentration (M) | 1.7 | 1.5 |
| Lutein productivity *^a^* (mg/L/day) | 3.68±0.44 | 2.86±0.43 |

*^a^* Values were averaged from three independent experiments (mean±SD).

Quadratic model in coded values (**Equation S1**)

*Y* = 2.86 – 0.024*x_1_* + 0.30*x_2_* + 0.51*x_3_* + 0.068*x_1_x_2_* – 0.12*x_1_x_3_* – 0.20*x_2_x_3_* – 0.76*x_1_*2 – 1.08*x_2_*2 – 1.37*x_3_*2 (**Equation S1**)

where *Y* is daily lutein productivity (mg/L/day), *x_1_* is blue LED percentage of total LEDs in coded unit, *x_2_* is KNO_3_ concentration in coded unit and *x_3_* is NaCl concentration in coded unit.
